# Biological and synthetic surfactant exposure increases antimicrobial gene occurrence in a freshwater mixed microbial biofilm environment

**DOI:** 10.1002/mbo3.1351

**Published:** 2023-03-17

**Authors:** Stephanie P. Gill, William J. Snelling, James S. G. Dooley, Nigel G. Ternan, Ibrahim M. Banat, Joerg Arnscheidt, William R. Hunter

**Affiliations:** ^1^ Department of Geography and Environmental Studies Ulster University Coleraine Londonderry Northern Ireland; ^2^ Nutrition Innovation Centre for Food and Health (NICHE) Ulster University Coleraine Londonderry Northern Ireland; ^3^ School of Biomedical Sciences Ulster University Coleraine Londonderry Northern Ireland; ^4^ Fisheries and Aquatic Ecosystems Branch Agri‐Food and Biosciences Institute Belfast Northern Ireland

**Keywords:** 16S rRNA, AMR gene, aquatic, biofilm, rhamnolipid, surfactant

## Abstract

Aquatic habitats are particularly susceptible to chemical pollution, such as antimicrobials, from domestic, agricultural, and industrial sources. This has led to the rapid increase of antimicrobial resistance (AMR) gene prevalence. Alternate approaches to counteract pathogenic bacteria are in development including synthetic and biological surfactants such as sodium dodecyl sulfate (SDS) and rhamnolipids. In the aquatic environment, these surfactants may be present as pollutants with the potential to affect biofilm formation and AMR gene occurrence. We tested the effects of rhamnolipid and SDS on aquatic biofilms in a freshwater stream in Northern Ireland. We grew biofilms on contaminant exposure substrates deployed within the stream over 4 weeks. We then extracted DNA and carried out shotgun sequencing using a MinION portable sequencer to determine microbial community composition, with 16S rRNA analyses (64,678 classifiable reads identified), and AMR gene occurrence (81 instances of AMR genes over 9 AMR gene classes) through a metagenomic analysis. There were no significant changes in community composition within all systems; however, biofilm exposed to rhamnolipid had a greater number of unique taxa as compared to SDS treatments and controls. AMR gene prevalence was higher in surfactant‐treated biofilms, although not significant, with biofilm exposed to rhamnolipids having the highest presence of AMR genes and classes compared to the control or SDS treatments. Our results suggest that the presence of rhamnolipid encourages an increase in the prevalence of AMR genes in biofilms produced in mixed‐use water bodies.

## Graphical abstract

Biofilms exposed to biological and synthetic surfactants may experience an increased prevalence of antimicrobial resistance genes. Of the nine total classes tested, eight were present in biofilm exposed to a biological surfactant, five were present after exposure to a synthetic surfactant, and four were present in our control.

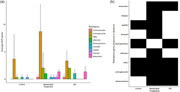

## 1 INTRODUCTION

Chemical contamination from point and nonpoint source pollution poses a major problem for our environment. As industries and the human population expand, more synthetic and anthropogenic toxic chemicals are released into the waterways from domestic, agricultural, and industrial sources (Fang et al., 2019; Yang et al., 2017). The continued presence of antimicrobials, heavy metals, and biocidal chemicals leads to an increasingly common selection for resistance genes, as the need for bacteria to protect themselves against a chemical hazard outweighs any cost of carrying the gene (Singer et al., 2016). Antimicrobial chemicals are used in medical environments for surface and device disinfection. Overuse of these chemicals has led to an increase in bacterial resistance to them and an increased frequency of antimicrobial resistance (AMR) genes appears to be associated with areas exposed to elevated concentrations of antimicrobial chemicals (Hartmann et al., 2016; Maillard, 2005). Based on metagenomic analyses AMR gene abundance is estimated to vary significantly across countries, with higher abundances more prevalent in areas with socioeconomic disadvantages (Hendriksen et al., 2019). Accurate attribution of global deaths to AMR remains a challenge. Recent estimates are at an approximate annual death toll of 4.95 million people and extrapolation of available data suggests further increases in the future (Dunachie et al., 2020; Murray et al., 2022).

Many microorganisms, including various bacterial taxa, preferentially reside within biofilms where they are protected from a variety of environmental stressors (Bernbom et al., 2011; DePas et al., 2014, Rode et al., 2020). Biofilms provide increased protection from antibiotics as the extracellular polymeric substance matrix surrounding biofilms limits antibiotic penetration (De Beer et al., 1994; Mah & O'Toole, 2001). Within this sheltered environment, horizontal gene transfer takes place which can increase the levels of AMR genes present within a biofilm in comparison to the planktonic state. Environmental biofilms are also common habitats of pathogenic bacteria and opportunistic pathogens such as *Escherichia coli, Legionella* spp., and *Pseudomonas aeruginosa* (Wingender & Flemming, 2011). Horizontal gene transfer of AMR genes can be facilitated by pathogenic bacteria residing within biofilms making them potential hotspots of AMR genes in the right conditions (Bowler et al., 2020; Ma et al., 2021).

Biosurfactants are naturally produced surfactants (chemicals that alter surface tension between liquids) from bacteria within a biofilm. They play a major role at the early stage of biofilm development, mainly through the maintenance of channels through the matrix which enhances the exchange of nutrients and gases with the ambient environment. Ultimately biosurfactants also result in the dissociation of biofilm surface layers thereby releasing sessile bacteria into planktonic forms (Banat et al., 2014; Marchant & Banat, 2012; Quinn et al., 2013). Although biosurfactants can naturally control biofilm development, and to some extent their structure, a high‐dosage application can also inhibit or eradicate biofilms (Satpute et al., 2016). In industrial and medical settings synthetic surfactants are typically used to remove unwanted biofilms; however, they also have found a large and increasing domestic use as essential ingredients in shampoos, soaps, toothpaste, and detergents (Ivanković & Hrenović, 2010). For a variety of medical and technical applications the use of surfactants, both alone and in combination with antibiotics, has been proposed to break down bacterial biofilms thereby reducing this reservoir of AMR genes (Wang et al., 2020; Zhang et al., 2016). However, growing awareness of the synthetic surfactants' environmental toxicity has focused more effort on the investigation of safer alternatives, such as biosurfactants (Paraszkiewicz et al., 2021). Rhamnolipid is a promising biosurfactant produced by bacteria of the genus *Pseudomonas* (Thakur et al., 2021). In combination with antibiotics, rhamnolipid has been shown to remove 95% of tested biofilms after exposure (Chen et al., 2019).

Research concerning the effects of surfactants and biosurfactants on biofilms and changes in abundance and diversity of AMR genes has hitherto only concerned industrial and medical biofilms, while environmental settings have received much less attention. In this study, we investigate how a biological surfactant, rhamnolipid, and a synthetic surfactant, sodium dodecyl sulfate (SDS), affect the development of microbial biofilms in a mixed land‐use stream environment. We aim to determine how exposure to both surfactants influenced microbial community composition and AMR gene prevalence of the exposed biofilms. We hypothesized that in a natural aquatic ecosystem, rhamnolipid and SDS exposure would alter biofilm community composition to favor taxa less sensitive to chemical exposure while also decreasing AMR gene abundance. Community composition changes were quantified through a 16S rRNA analysis. AMR gene abundances were examined through metagenomic analysis of resistance to elfamycin, aminoglycoside, tetracycline, isoniazid, aminocoumarin, fluoroquinolone, and rifampin.

## 2 EXPERIMENTAL PROCEDURES

### 2.1 Experimental setup

The Ballysally Blagh is a second‐order lowland stream at the town of Coleraine near the North Coast of Northern Ireland, UK. This tributary to the lower River Bann has an average mainstream channel slope (S1085) of 6.22 m km^−1^ between points of 10% and 85% of mainstream length above the catchment outlet (Gardner & Wilcock, 2003). It drains a 14.2 km^2^ catchment of water gley soils with mixed land cover, that is, grassland 55.9%, arable/horticultural 21.9%, bog 13.7%, urban 7.3%, woodland <2% (National River Flow Archive, 2022). The nonurban area is predominantly used for agricultural purposes (McClean & Hunter, 2020). The Ballysally Blagh's baseflow index of 0.51 characterizes an intermediate level of water transfers toward its stream flow from storage in superficial deposits of mixed permeability. Artificial drainage and stream channel profiling have resulted in a flashy stream discharge response to precipitation as indicated by the stream's Q_5_:Q_95_ discharge ratio of 24 (National River Flow Archive station 203050).

Nine contaminant exposure substrate (CES) cups were prepared as per Costello et al. (2016) using glass fiber (GF/C) filters. Clean tea filters (Lipton polypropylene nonwoven mesh (<0.25 mm) [Mori et al., 2021]) were added into each lid as a particulate sieve to minimize sedimentation on the glass fiber filters. Three cups contained agarose gel dosed with 150 ppm rhamnolipid (JBR 425, Jeneil Biosurfactant Company), three cups contained agarose gel dosed with an equimolar concentration of SDS (75 ppm) (BDH, AnalaR) and the final three cups were controls with agarose gel only. A rhamnolipid concentration of 150 ppm was used as it was established to be quite effective in previous studies (Gill, Hunter, et al., 2022; Gill, Kregting, et al., 2022). Cups were zip‐tied together and attached to a large, submerged metal bar to secure their position on the stream bed of the Ballysally Blagh at 55°08′44.4″N 6°40′19.0″W.

### 2.2 Sample collection

CES cups were deployed in the stream for a period of 4 weeks from February 2020 to March 2020. To account for air contamination, three additional GF/C filters were used when CES cups were collected and exposed to air bacteria at the collection site for the whole duration of cup collection, approximately 30 min. These three filters were then treated identically to all other filters from the experiment for the rest of the study to allow for the identification of any potential external microbial contaminants that were not from the stream environment. All cups were rinsed in ultrapure water to remove any debris that collected on top of each cup and filter. Individual sterile tweezers were then used to remove the GF/C filters with developed biofilm from the CES cups under a sterile laminar flow hood. Filters were placed immediately into sterile test tubes, sealed, and frozen at −20°C.

DNA extractions were performed using a FastDNA Spin kit for Soil (MP Biomedical) as per the manufacturer's guidelines. Biofilm material was scraped off the filters and recovered from the stream before performing DNA extractions. In the absence of visible biofilm on the air controls, the actual filters were processed to obtain any DNA that may have originated from contamination. To confirm DNA presence within each sample, DNA was quantitated using an ND1000 Nanodrop Spectrophotometer (Labtech Int). Shotgun sequencing was performed with a MinION portable sequencer and R9 flow cell (Oxford Nanopore), in accordance with the manufacturer's guidelines, including the Agencourt AMPure XP Bead (Beckman‐Coulter) clean‐up step, using a Rapid Barcoding Sequencing kit SQK‐RBK004 (Oxford Nanopore) with a minimal acceptable DNA quality score of 7. Similar DNA yields were used for sequencing to allow for appropriate comparison between samples, with each of the 12 samples analyzed in triplicates that were then averaged. All samples from the main experiment, except for one low‐yield experimental control, yielded 280 ng of DNA. Air controls and the single low‐yield experimental control yielded 48 ng of DNA. Results from the low‐yield experimental control and the high‐yield experimental control samples were compared before being averaged later in the analysis protocol to ensure similar species abundance ratios occurred.

### 2.3 Data analysis

The twelve resultant Fast Q files from sequencing were processed individually following similar methods as Solcova et al. (2021) and Zhou et al. (2022). The 16S rRNA workflow from Fastq WIMP (v2021.03.05) software was utilized to obtain bacterial species identification. Fastq Antimicrobial Resistance (v2021.05.17) software was used separately for the identification of AMR gene prevalence as well as changes to efflux pumps conferring antibiotic resistance, and peptide antibiotic resistance genes (Oxford Nanopore). Recommended settings were used along with a minimum DNA quality score of 7 to obtain higher‐quality sequences, where all 326,805 sequences used had an average quality score of 10. Results from both software packages were downloaded as CSV files for further analysis in R statistical software. Any bacterial species in the air control samples identified with the Fastq WIMP 16S rRNA workflow were removed from all files before further analyses continued based on the assumption that these were contaminating species of airborne bacteria.

Statistical analysis employed R version 4.0.3 (R Core Team, 2020) and the R packages “car” for normality testing (Fox & Weisberg, 2018), “vegan” for univariate and multivariate statistics (Oksanen et al., 2020), and “ggplot2” for figure creation (Wickham, 2016). Past 3 software (Hammer et al., 2001) was used to perform all nonmetric multidimensional scaling (NMDS) analyses. All univariate data met assumptions of normality and homogeneity of variance. Significance was assumed at *p* < 0.05. Diversity was first examined in the 16S rRNA results using the Shannon–Wiener diversity index (examining the abundance and evenness of species present). We included all singletons and doubletons in this to account for potentially rare species. Diversity index values were analyzed with a one‐way analysis of variance (ANOVA) test to determine if any diversities were significantly different from each other (*p* < 0.05). Singletons and doubletons were then removed, and data were rarified and subsampled. This action allowed us to account for the differences in DNA yields within our data by keeping the same ratio of organisms within each sample but reducing abundance where there was an equal amount of total taxa in each sample. An NMDS was then performed followed by an analysis of similarities (ANOSIM) to test for significant differences among the community composition of biofilms.

AMR gene presence, that is, frequency of presence or absence of each gene as noted by the Fastq Antimicrobial Resistance software, was examined first with an NMDS and then a permutational multivariant analysis of variance (PERMANOVA). This determined if there were differences among both frequency of presence of detected AMR genes and the types of genes. We then ran an ANOVA to identify significant differences in only the types of genes present.

## 3 RESULTS

### 3.1 Community composition

First, we determined how the community composition of biofilms shifted after exposure to both SDS and rhamnolipids. Overall, we found large dominance by *Escherichia*, *Acidovorax, Janthinobacterium*, *Shigella*, *Flavobacterium*, and *Pseudomonas* spp. in all treatments and controls. The rhamnolipid‐treated samples contained 566 unique taxa, SDS treatments had 478, while control treatments had the lowest at 386. However, despite having the lowest total taxonomic richness, the controls still had 57 unique taxa when compared to the treatments. Rhamnolipid had 177 unique taxa and SDS had 96.

**Figure 1:**
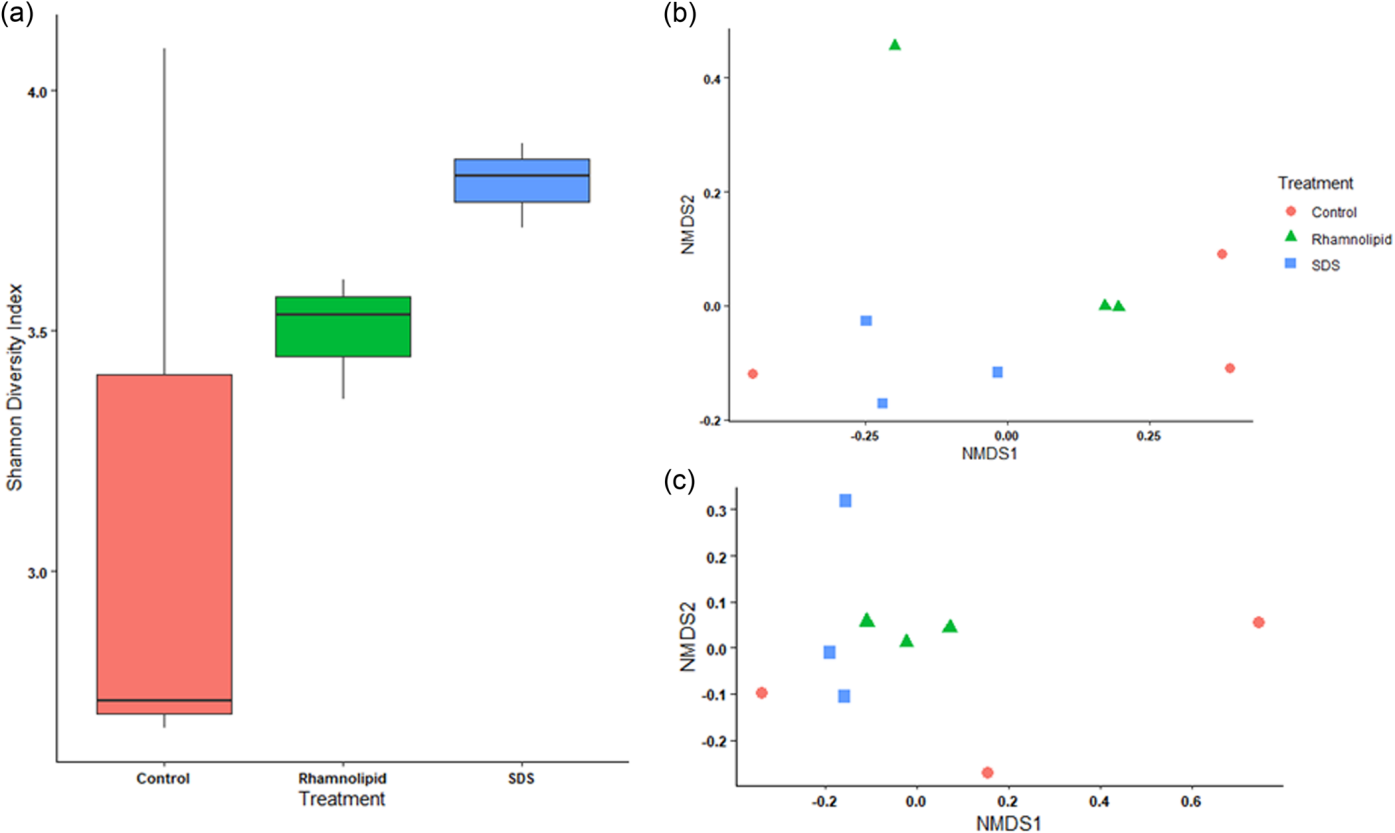
Microbial diversity with the (a) Shannon–Wiener diversity index and two nonmetric multidimensional scaling analyses depicting community differences of naturally grown microbial biofilms continuously exposed to 150 ppm of the biosurfactant rhamnolipid and 75 ppm of the synthetic surfactant sodium dodecyl sulfate (SDS) for 4 weeks in a stream with a mixed land use catchment (b) with and (c) without the top 10 most dominant genera identified. These mixed biofilms were naturally developed using contaminant exposure substrate cups.

We found no significant differences in microbial diversity among any of the biofilms (ANOVA *F* = 1.409, *p* = 0.315) (Figure 1a). Although biofilm in SDS treatments had higher taxonomic diversity than in rhamnolipid treatments, the control samples had the largest range of diversity, accounting for both the highest and lowest diversity overall. Consequently, while there were some differences in the diversity arising in SDS and rhamnolipid treatments, neither treatment appeared significantly different from the control. The employed diversity measurements examine both abundance and evenness, implying that although rhamnolipid treatments contained a greater number of unique taxa, most likely that many of these were low abundance taxa which reduced the treatment's overall diversity score.

NMDS was used to identify differences in the overall community composition of treatment groups, which would demonstrate if exposure to either chemical had altered biofilm composition. There were no distinct groupings within the NMDS (Figure 1b), which was confirmed by an ANOSIM (*R* = 0.1512, *p* = 0.226), implying a lack of unique community compositions within the biofilms. To identify if there were differences among the rarer taxa within our samples, the NMDS (Figure 1c) and ANOSIM (*R* = 0.1111, *p* = 0.14) were rerun without the top 10 most dominant taxa and no significant differences or groupings were identified within the rarer taxa across the treatments and controls.

**Figure 2:**
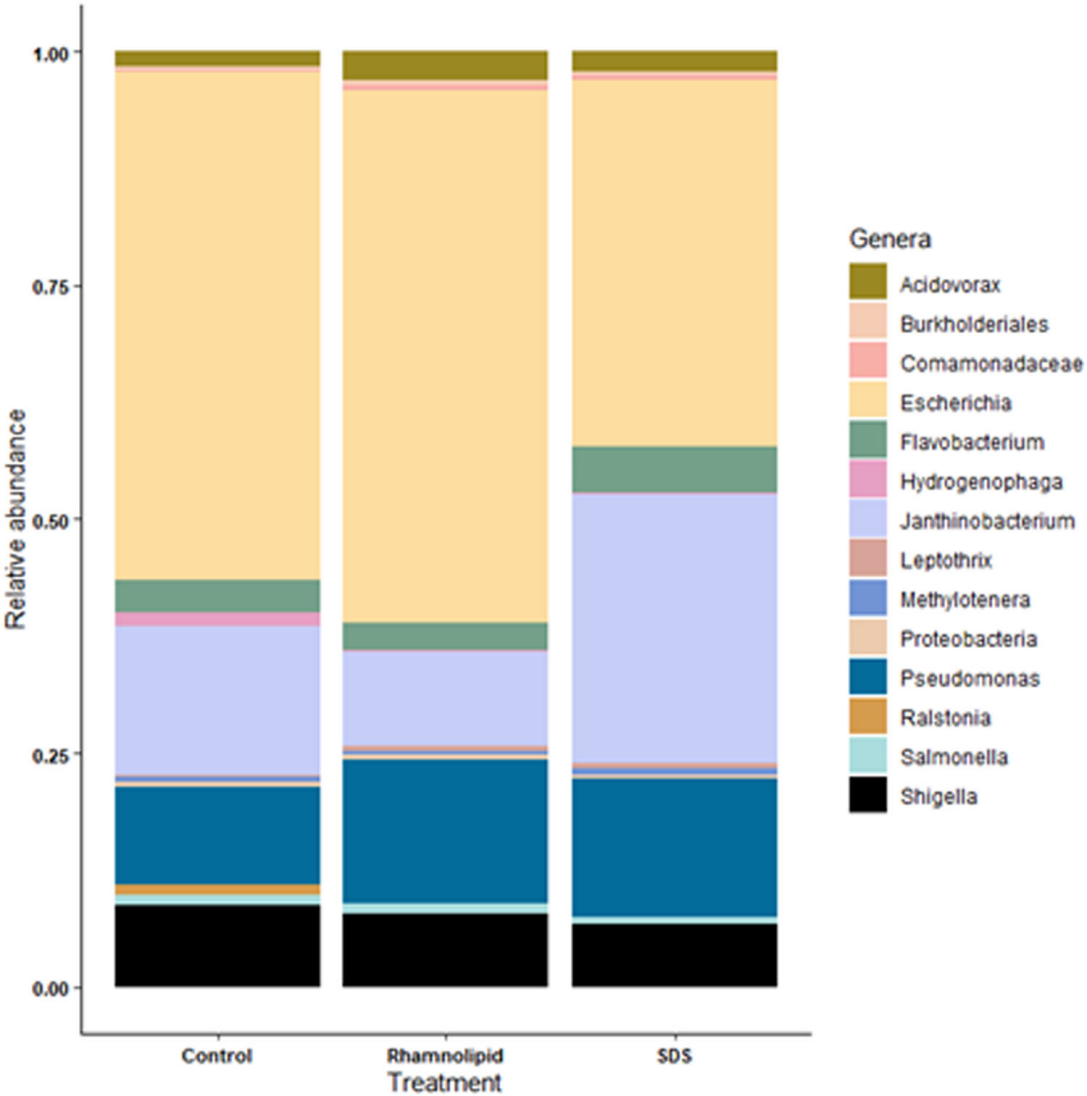
Relative abundances of the most dominant genera, as measured by sequence abundance, within microbial biofilm communities from mixed biofilms grown in contaminant exposure substrate cups and continuously exposed to 150 ppm of the biosurfactant rhamnolipid and 75 ppm of the synthetic surfactant sodium dodecyl sulfate (SDS) for 4 weeks in a stream with a mixed land use catchment.

Dominant taxa were also similar among treatments. All treatments and controls were dominated by *Escherichia*, *Acidovorax, Janthinobacterium*, *Shigella*, *Flavobacterium*, and *Pseudomonas* spp. Except for *Ralstonia* and *Leptothrix*, where the former was present only in the control, while the latter was present only in the rhamnolipid and SDS treatments, lists of the top 10 genera identified within each treatment and control were almost identical (Table 1). While the former two genera were not among the top 10 for all groups, they were still present in each control or treatment albeit sometimes at lower abundances. Although there was considerable overlap in dominant genera within each treatment, the abundance of taxa identified (based on 16S rRNA sequences) within each of the genera differed (Figure 2). Rhamnolipid treatments had the greatest number of *Escherichia*, while the control had the greatest abundance of *Shigella*, and SDS had the greatest abundance of *Janthinobacterium*. For several genera such as *Escherichia, Janthinobacterium*, and *Shigella* the control and rhamnolipid treatments had more comparable abundances than the rhamnolipid and SDS treatments. Rhamnolipid and SDS treatments had similar *Pseudomonas* abundances when compared to the control. Therefore, it is reasonable to infer that each genus had its sensitivity, and *Shigella* spp. may be the most sensitive to chemical contamination as compared to *Escherichia* or *Janthinobacterium*.

**Table 1:** Top 10 classifiable genera within microbial biofilms continuously exposed to 150 ppm of the biosurfactant rhamnolipid and 75 ppm of the synthetic surfactant sodium dodecyl sulfate (SDS) for 4 weeks in a stream.

	Treatment		
	Rhamnolipid	SDS	Control
Top classifiable genera	*Escherichia*	*Escherichia*	*Escherichia*
	*Pseudomonas*	*Janthinobacterium*	*Janthinobacterium*
	*Janthinobacterium*	*Pseudomonas*	*Pseudomonas*
	*Shigella*	*Shigella*	*Shigella*
	*Acidovorax*	*Flavobacterium*	*Flavobacterium*
	*Flavobacterium*	*Acidovorax*	*Acidovorax*
	*Salmonella*	*Salmonella*	*Hydrogenophaga*
	*Leptothrix*	*Methylotenera*	*Ralstonia*
	*Methylotenera*	*Leptothrix*	*Salmonella*
	*Hydrogenophaga*	*Hydrogenophaga*	*Methylotenera*

*Note*: Biofilms were mixed and naturally developed using contaminant exposure substrate cups. Genera are listed in order of dominance within each treatment.

### 3.2 Antimicrobial resistance genes

**Figure 3:**
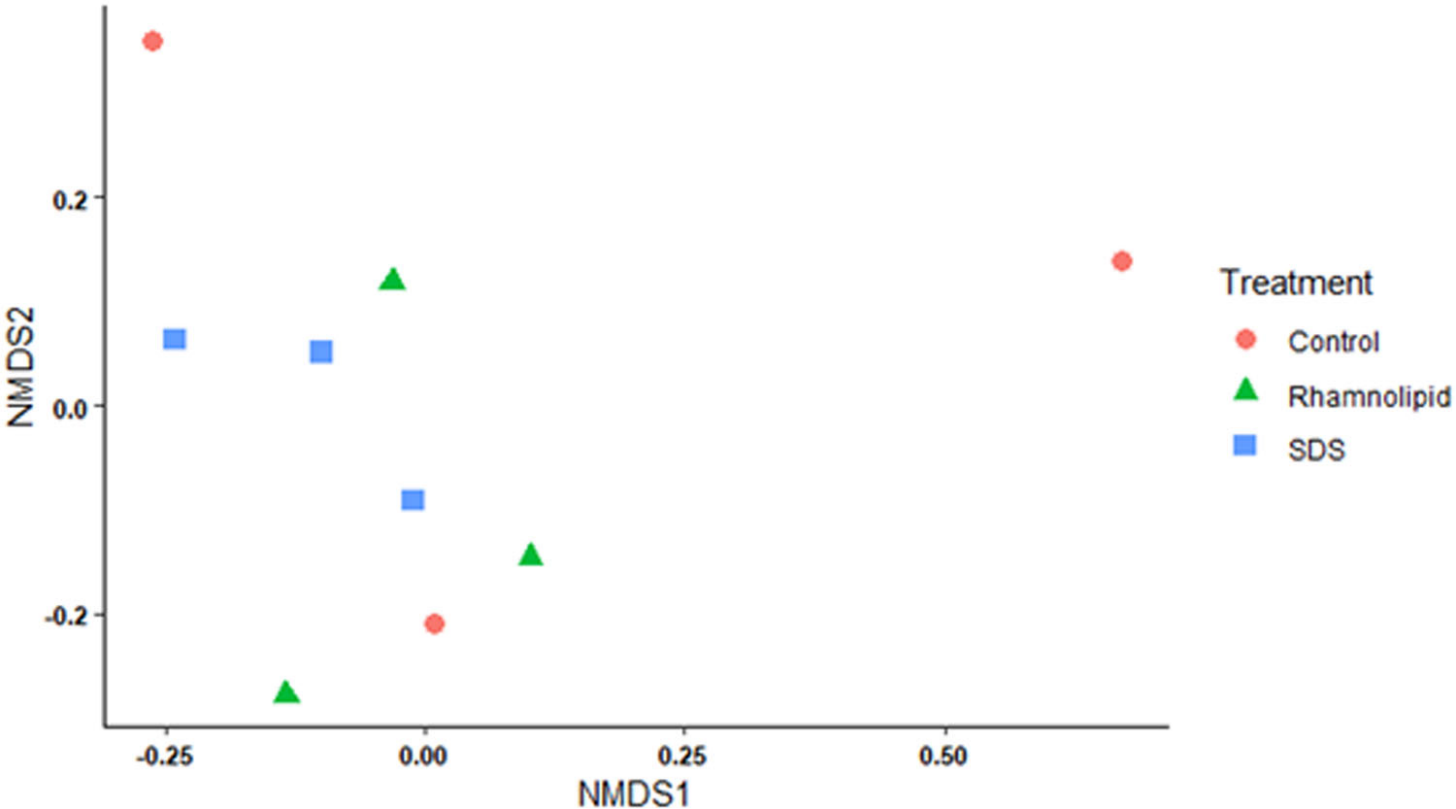
Nonmetric multidimensional scaling depicting the differences in antimicrobial resistance genes identified within mixed microbial biofilms grown in contaminant exposure substrate cups and continuously exposed to 150 ppm of the biosurfactant rhamnolipid and 75 ppm of the synthetic surfactant sodium dodecyl sulfate (SDS) for 4 weeks in a stream with a mixed land use catchment.

**Figure 4:**
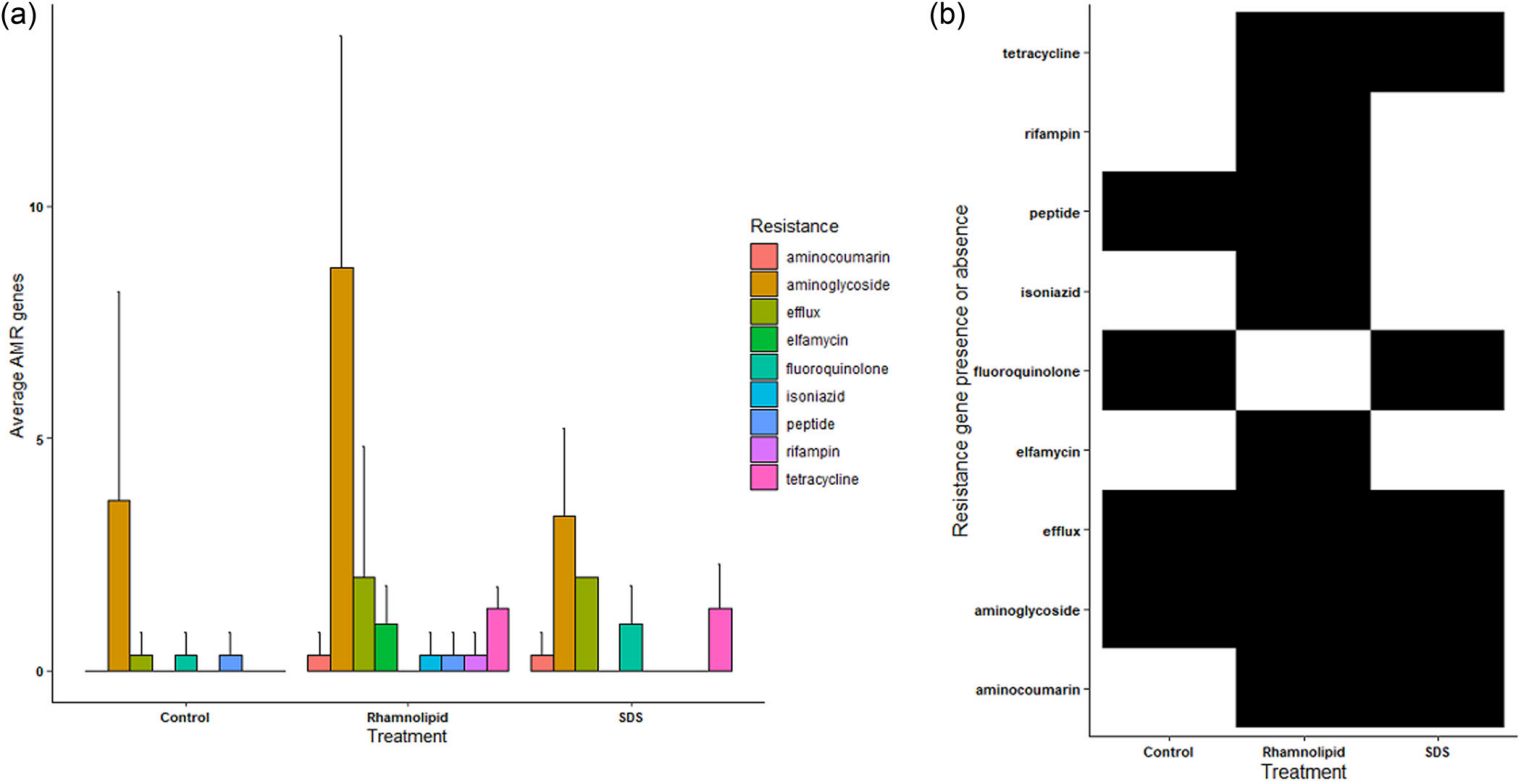
Differences in (a) the average number of antimicrobial resistance (AMR) genes present and (b) the type of AMR genes present, where black signifies presence and white signifies absence, within a mixed microbial biofilm grown in contaminant exposure substrate cups continuously exposed to 150 ppm of the biosurfactant rhamnolipid and 75 ppm of the synthetic surfactant sodium dodecyl sulfate (SDS) for 4 weeks in a stream with a mixed land use catchment.

There were no statistically significant differences in the abundance of AMR genes within each treatment (PERMANOVA *F* = 1.3812, *p* = 0.195). This was confirmed by NMDS as there were no distinct groupings among treatments and controls (Figure 3). However, further examination of the differences in AMR genes showed generally higher numbers of AMR genes within the rhamnolipid‐treated cups when compared to the SDS treatments and the controls (Figure 4a), although the difference was not significant (ANOVA *F* = 1.926, *p* = 0.226). The presence of each AMR gene was also not significantly different across treatments (MANOVA: elfamycin *F* = 3, *p* = 0.125; aminoglycoside *F* = 1.1, *p* = 0.392; tetracycline *F* = 1.78, *p* = 0.113; isoniazid *F* = 1, *p* = 0.422; efflux pumps *F* = 0.676, *p* = 0.544; aminocoumarin *F* = 0.5, *p* = 0.629; fluoroquinolone *F* = 1.75, *p* = 0.252; rifampin *F* = 1, *p* = 0.422; peptide *F* = 0.5, *p* = 0.629). Overall 81 instances of AMR gene presence were identified across nine AMR classes. The rhamnolipid treatment was the only treatment in which genes encoding elfamycin, isoniazid, and rifampin resistance were detected, and it also had the highest abundance of aminoglycoside resistance genes. Tetracycline and aminocoumarin resistance were also only detected in treatments, but not in the controls (Figure 4b).

## 4 DISCUSSION

This study aimed to determine how rhamnolipid and SDS exposure affects the occurrence of AMR genes in natural mixed biofilms. Although the application of rhamnolipid and SDS treatments did not affect overall biofilm diversity and community composition there were clear visual differences in AMR gene prevalence. However, these were not statistically significant, probably due to the small sample size employed in the study. The unique presence of AMR genes was only identified after exposure to rhamnolipid with elfamycin, isoniazid, and rifampin resistance. Tetracycline and aminocoumarin resistances were also identified in biofilms after exposure to either surfactant which may be problematic as surfactants are used to enhance antibiotic potency.

The Ballysally Blagh drains a catchment that is 92.7% agricultural and 7.3% urban land (Hunter et al., 2021; McClean & Hunter, 2020). As such, it regularly receives significant inputs of fecal material as a consequence of agricultural slurry spreading, and episodically from a combined sewer overflow at a small sewage pumping station located upstream of the sampling site. A wet February (right after the closed season for slurry application had ended) would also imply that extra fecal material from an initial slurry application on waterlogged soil and wastewater from the sewer overflow was present in the waterways. The Ballysally Blagh's microbial community was dominated by *Escherichia*, *Acidovorax, Janthinobacterium*, *Shigella*, *Flavobacterium*, and *Pseudomonas* spp. within it, along with several other similar genera. *Escherichia* (Carlos et al., 2010) and *Shigella* (Olalemi & Dauda, 2018) are commonly used as markers of fecal contamination in waterways from both human and animal sources. *Pseudomonas* and *Salmonella* bacterial presence are also used as an alternate indicator of fecal contamination due to their high presence in contaminated waters (Januário et al., 2019; Liang et al., 2015). All dominant genera are found in agricultural and urban environments (Cabral, 2010; Rosi et al., 2018), with some of them already developed resistance to antibiotic and chemical exposure (Kumar et al., 2019; Pang et al., 2019; Saraceno et al., 2021). *Pseudomonas* and *Acidovorax* spp. are extremely adept at survival in polluted environments, for example, also dominating once‐diverse habitats after chlorine exposure (Jia et al., 2015).

The lack of differences identified in the community composition between treatments is likely to be caused by the domination of these bacterial taxa, probably due to the stream being highly exposed to inputs from agricultural and urban land. Over 1/3 of the present taxa within our treatments and controls were made up of the same genera, and only two genera *Ralstonia* and *Leptothrix* were found in unique treatments. The greater dominance of *Ralstonia*, a potential plant pathogen (Wei et al., 2018), within the control treatment may indicate greater sensitivity to chemical exposure. Alternatively, the greater concentrations of *Leptothrix* within the surfactant treatments may indicate lower chemical sensitivity. *Escherichia* growth was also fairly limited within SDS‐treated systems when compared to the control and rhamnolipid treatments and *Janthinobacterium* growth was increased, potentially indicating less sensitivity of *Janthinobacterium* to SDS.

More taxa were identified within the rhamnolipid and SDS treatments as compared to the control. Typically, exposure to either chemical has led to a reduced taxa count (Gill, Hunter, et al., 2022). In our experiments, exposure to rhamnolipids yielded the highest number of unique taxa, SDS the second highest, and the control had the lowest number; the same pattern was replicated for AMR gene abundance and diversity. Unique taxa included *Stella*, a star‐shaped bacterium commonly found in sewage and soil/freshwater environments (Vasilyeva, 1985) which was observed in the rhamnolipid treatment only, and *Haliangium*, a bacterial genus previously found in wastewater treatment plant sludge (Gao et al., 2016) which was observed in the SDS treatments only. Although these taxa were unique to their treatments, they are commonly found in environments exposed to sewage and such conditions tend to facilitate horizontal gene transfer and increase the abundance of AMR genes (Uluseker et al., 2021). We propose the possibility that surfactants may enhance the passage of AMR genes into bacterial cells. Surfactants, such as SDS, can disrupt cell membranes (Shehadul Islam et al., 2017), potentially making the uptake of genes from the environment, possibly through horizontal gene transfer, easier. However, we also emphasize that as there were taxa unique to the control samples, the presence of either surfactant may also make the habitat more inhospitable for some taxa.

Treatments and controls, all of them, acquired AMR genes during the experiment, which is to be expected considering the high levels of resistance typically found in *Escherichia* and *Pseudomonas* spp. (Pang et al., 2019; Tadesse et al., 2012) and the high likelihood of fecal pollution in our agricultural and urban streams. Previous research has identified *Escherichia* and *Pseudomonas* resistance to aminoglycosides (Garneau‐Tsodikova & Labby, 2016), fluoroquinolones (Redgrave et al., 2014), and tetracyclines (Gasparrini et al., 2020) among others. Rhamnolipid‐treated systems developed a greater abundance of AMR genes and AMR gene classes when compared to the control or SDS treatments, and they were also the systems with the highest concentrations of *Escherichia*. While the differences in types of AMR gene classes and AMR gene abundance are visible (Figure 4), they are not statistically significant. This is likely to be a consequence of the small sample size within our study, which limits the power of inferential statistical tests (Underwood, 1997). Based on our data it is, thus, reasonable to suggest that rhamnolipid exposure may promote the retention of AMR genes within a biofilm and potentially encourage the growth of pathogenic strains of bacterial genera such as *Escherichia*. They were also the only treatments to affect AMR gene prevalence with *Streptomyces* (which had a high prevalence of elfamycin resistance), and *Mycobacterium* (which had a high prevalence of isoniazid resistance), and the only treatment to develop resistance to rifampin. This is potentially problematic given the interest in rhamnolipids' ability to enhance antibiotic effects and antibiotic potency (Bharali et al., 2013; Chen et al., 2019).

Surfactants in medical (Deo et al., 2010) and industrial applications (Schlüter et al., 2007) have also been investigated for their ability to reduce the number of antibiotic‐resistant bacteria in hospital and care environments and wastewater treatment plants. It has also been noted that bacteria have started to develop resistance to surfactants, specifically quaternary ammonium compounds (QACs), and that bacterial strains with QAC resistance are more likely to co‐select for antibiotic resistance as well (Gaze et al., 2005). While SDS and rhamnolipids are not QACs, our data set suggests that there is a larger abundance and diversity of AMR genes developing among biofilm able to grow on an exposed substrate. Our results suggest that rhamnolipids may have an impact on the abundance of AMR genes in the wild and that SDS may also slightly encourage AMR gene selection. It is likely that although biosurfactants and surfactants may facilitate access of antibiotics to bacterial cells through weakening cell membranes, the action of combined treatments also potentially promotes increased selection for AMR genes among surviving bacteria.

We sought to identify the effects of a surfactant and biosurfactant on the community composition and AMR gene abundance of a mixed microbial biofilm grown in a stream exposed to agricultural and urban sources of pollution. Based on previous research we assumed that biofilm composition would alter after exposure to rhamnolipid and SDS and that there would be fewer AMR genes present (Chen et al., 2019; Wang et al., 2020; Zhang et al., 2016). However, we observed that only minimal compositional and diversity changes occurred, and a nonsignificant increase in AMR gene abundance after exposure. Likely, an environment with a substantial presence of *Escherichia*, *Acidovorax, Janthinobacterium*, *Shigella*, *Flavobacterium*, and *Pseudomonas* favored resilient bacteria, allowing for biofilm compositional dominance by just a few species. These opportunistic pathogens are also well known for their selection of AMR genes in the presence of antibiotics and antimicrobial chemicals, which may have been encouraged by the presence of rhamnolipid and to a lesser extent of SDS. This would be extremely unfortunate as rhamnolipids are currently being studied for their ability to increase antibiotic potency in controlled experiments (Juma et al., 2020).

Our study is the first example of the biosurfactant rhamnolipid increasing AMR gene abundance within a biofilm. These observations, however, require confirmation through larger‐scale field experiments, supplemented by in vitro studies to elucidate the biochemical mechanisms under controlled laboratory conditions. This approach is necessary to accurately determine the impact of introducing rhamnolipids to agricultural and urban waterways, and the implications for environmental management. If biosurfactants, such as rhamnolipid, consistently increased the abundance and diversity of AMR genes in natural biofilms then their presence in the aquatic ecosystem could be detrimental to both human and animal health as they could facilitate the bacterial acquisition of multi‐resistance against antibiotics, thus encouraging the evolution of new multi‐resistant organisms.

## AUTHOR CONTRIBUTIONS

**Stephanie P. Gill**: Conceptualization (lead); data curation (equal); formal analysis (lead); funding acquisition (equal); investigation (lead); methodology (equal); project administration (equal); resources (equal); software (equal); validation (equal); visualization (equal); writing—original draft (lead); writing—review and editing (equal). **William J. Snelling**: Conceptualization (lead); data curation (equal); formal analysis (equal); funding acquisition (equal); investigation (equal); methodology (equal); resources (lead); software (lead); validation (equal); visualization (equal); writing—original draft (supporting); writing—review and editing (equal). **James S. G. Dooley**: Conceptualization (equal); data curation (equal); formal analysis (equal); funding acquisition (equal); investigation (equal); methodology (equal); project administration (equal); resources (equal); software (equal); supervision (equal); validation (equal); visualization (equal); writing—original draft (supporting); writing—review and editing (equal). **Nigel G. Ternan**: Conceptualization (equal); data curation (equal); formal analysis (equal); funding acquisition (equal); investigation (equal); methodology (equal); project administration (equal); resources (equal); software (equal); supervision (equal); validation (equal); visualization (equal); writing—original draft (supporting); writing—review and editing (equal). **Ibrahim M. Banat**: Conceptualization (equal); data curation (equal); formal analysis (equal); funding acquisition (equal); investigation (equal); methodology (equal); project administration (equal); resources (equal); software (equal); supervision (equal); validation (equal); visualization (equal); writing—original draft (equal); writing—review and editing (equal). **Joerg Arnscheidt**: Conceptualization (equal); data curation (equal); formal analysis (equal); funding acquisition (equal); investigation (equal); methodology (equal); project administration (equal); resources (equal); software (equal); supervision (equal); validation (equal); visualization (equal); writing—original draft (equal); writing—review and editing (equal). **William R. Hunter**: Conceptualization (equal); data curation (equal); formal analysis (equal); funding acquisition (equal); investigation (equal); methodology (equal); project administration (equal); resources (equal); software (equal); supervision (lead); validation (equal); visualization (equal); writing—original draft (equal); writing—review and editing (equal).

## CONFLICT OF INTEREST STATEMENT

None declared.

## ETHICS STATEMENT

None required.

## Data Availability

Data files are available in Zenodo at https://doi.org/10.5281/zenodo.7079136.
